# Midazolam-Induced Seizure-Like Activity in Five Neonates: A case series

**DOI:** 10.18295/squmj.6.2024.030

**Published:** 2024-08-29

**Authors:** Heba Shaker, Fatema Al-Amrani, Hilal Al Mandhari

**Affiliations:** Child Health Department, Sultan Qaboos University Hospital, Sultan Qaboos University, Muscat, Oman

**Keywords:** Midazolam, Intravenous Injection, Seizures, Newborn Infant, Hypnotics and Sedatives, Case Report, Oman

## Abstract

An intravenous (IV) administration of midazolam may result in seizure-like activity or movement. This report describes 5 neonates who developed seizure-like movements after IV midazolam injection. The patients presented between 2019 and 2022 and were admitted to a neonatal intensive care unit located within an academic centre in Muscat, Oman. The abnormal movements occurred shortly after IV bolus administration of midazolam. None of the patients experienced seizure-like movements after receiving midazolam infusions. The seizure-like movements were aborted either spontaneously or by antiseizure medications. In addition, seizure recurrence was not observed in any of the infants during the later stages of their treatment. Since this adverse effect might be related to the speed of the bolus administration, IV midazolam must be given as a slow bolus over 2–3 minutes followed by a slow flush of normal saline. To prevent midazolam’s potential adverse effect on newborns, neonatal caregivers must be aware of it.

Midazolam is a short-acting benzodiazepine with a rapid onset of action and a short half-life.^1^ It is used frequently in the neonatal intensive care unit (NICU) for the treatment of seizures, procedural sedation and sedation in ventilated infants.^2^ The effects of midazolam are produced by binding to neurotransmitter receptors activated by gamma-aminobutyric acid (GABA). GABA-A receptors are responsible for most of the central nervous system’s (CNS) inhibitory neurotransmission. Benzodiazepines act on GABA-A receptors by binding to a specific site.^3^ Since infants may experience moderate to severe pain, agitation and irritability in the NICU, using sedation to keep them comfortable during painful medical interventions is useful.^4^

Midazolam has several serious known adverse reactions such as respiratory depression and hypotension.^3^ Other side effects known as paradoxical reactions to midazolam are, for example, hyperexcitability, restlessness and myoclonic jerks. Seizures or seizure-like activity have been reported following rapid bolus administration of midazolam.^1^^,^^5^^,^^6^ Seizure-like activity or myoclonic jerks are rare side effects of midazolam. We report 5 cases of seizure-like abnormal movements in neonates with the aim of raising awareness among neonatologists and other neonatal health practitioners of this rare adverse reaction to midazolam.

All neonates described in this study were admitted to a level III-IV NICU located within an academic centre, with perinatal services and an average birth rate of 5,000 births per year. This NICU is also a referral unit for complex neonatal cases from other secondary or peripheral hospitals. Approximately 450–500 neonates of varying gestational ages are admitted per year to this NICU. Midazolam at a dose of 0.1 mg/kg/dose is sometimes used for procedural sedation and the management of agitation for mechanically ventilated neonates. Before administration, midazolam is diluted in normal saline by adding 5 mg of midazolam to 4 mL of normal saline to produce a 1 mg:1 mL dilution. It is normally administered by the bedside nurse intravenously as a slow bolus, followed by a slow 2 mL flush of normal saline.

## CASE 1

The first case is a newborn male infant born at 37 weeks of gestation, weighing 2,540 g, to non-consanguineous parents to a G4P3 mother by caesarean section in 2021. The mother had late latent syphilis, treated with penicillin G. The infant was shifted to the NICU for a workup to exclude congenital syphilis. He was admitted in a stable condition in room air and the physical examination was unremarkable. The syphilis screening serology was positive, but the rapid plasma reagent was negative. On day 2 of life, midazolam 0.1 mg/kg was given for procedural sedation before a lumbar puncture (LP). The dose was given intravenously through a peripheral cannula, as a slow bolus, followed by a 2 mL flush of normal saline pushed slowly, before the procedure. The infant had stable vital signs (heart rate = 133 beats/minute, respiratory rate = 55 breaths/minute, oxygen saturation = 99%, mean blood pressure [MBP] = 59 mmHg). During the LP, he had clonic jerks of the upper limbs, therefore the procedure was aborted. He received a loading dose of IV levetiracetam 20 mg/kg because the abnormal movements continued for more than 5 minutes. However, the abnormal movements continued after the levetiracetam loading, so a half-loading dose of levetiracetam 10 mg/kg was given after which the abnormal movements stopped. His vital signs, after the event, were a heart rate of 128 beats/minute, respiratory rate of 34 breaths/minute, oxygen saturation of 97% and MBP of 63 mmHg. A septic workup was performed, and as it was negative, he received cefotaxime and acyclovir for 72 hours. Treponema pallidum haemagglutination was positive with a titre of 640. Syphilis IgM was <0.90 (i.e. negative). The brain ultrasound was normal. A routine electroencephalogram (EEG) was done after the episode and showed no epileptic discharges. The baby received penicillin G for 10 days. The seizure-like movement did not recur, and at follow-up at 6 months of age, no seizures or neurodevelopment deficits were documented.

## CASE 2

The second case study is a newborn male neonate, late preterm at 36^4/7^ weeks of gestational age, born in 2022 to a G7P3A3 mother with gestational diabetes, on metformin. He was born via normal vaginal delivery with Apgar scores of 6 and 9 at 1 and 5 minutes, respectively, and a birth weight of 3,320 g. He was admitted to the NICU with respiratory distress, presumed neonatal sepsis, hypoglycaemia and right shoulder dystocia. He was placed on a high-flow nasal cannula (HFNC) for 48 hours and then weaned to room air. Antibiotics were commenced for suspected neonatal sepsis. On the 10th day of life, a LP was planned and the infant received IV midazolam 0.1 mg/kg for procedural sedation. The dose was given through a peripheral cannula as a slow bolus followed by a flush of 2 mL of normal saline. His vital signs were stable (heart rate = 155 beats/minute, respiratory rate = 34 breaths/minute, oxygen saturation = 98%, MBP = 71 mmHg). While doing the LP, he immediately developed clonic movements of his 4 limbs that lasted for 2 minutes, associated with apnoea and grimacing. After the event, his vital signs were: heart rate = 200 beats/minute, respiratory rate = 28 breaths/minute, oxygen saturation = 88%, MBP = 66 mmHg; during the LP, the infant was on room air, but after the procedure, he required a HFNC. Antibiotics were upgraded to meningitis doses and acyclovir was started. The EEG was normal with no epileptic discharges. The herpes simplex virus polymerase chain reaction (PCR), and varicella zoster PCR were negative, so acyclovir was stopped. The neonate received antibiotics for 7 days and he was discharged on day 16 of life. He had no recurrence of abnormal movements. A follow-up in the clinic at 5 months of age showed normal growth and development, and the neurological examination was normal.

## CASE 3

The third case is an extreme preterm male neonate born at 25 weeks gestational age in 2019 to a G4P3 mother who received dexamethasone and antibiotics. The infant was born via caesarean section because of a breech presentation and cord prolapse; he had a birthweight of 800 g and Apgar scores 8 and 9 at 1 and 5 minutes, respectively. He was intubated at birth and was given 1 dose of endotracheal surfactant. His NICU course was complicated with stage II necrotising enterocolitis at the post-menstrual age of 34 weeks. The blood culture was reported positive for methicillin-resistant staphylococcus aureus. On day 69 of life, an LP was arranged to evaluate for meningitis. He was given midazolam intravenously (0.1 mg/kg) for procedural sedation before the LP. The dose was given through a peripheral cannula as a slow bolus, followed by a flush of 2 mL of normal saline. His vital signs before the procedure were: heart rate = 129 beats/minute, respiratory rate = 55 breaths/minute, oxygen saturation = 100% and MBP = 50 mmHg. Shortly after the administration of the dose, the infant developed clonic seizure-like movements of the 4 limbs, lasting for 2 minutes. He was loaded with levetiracetam. His vital signs after the abnormal movements were: heart rate = 174 beats/minute, respiratory rate = 49 breaths/minute, oxygen saturation = 96% and MBP = 45 mmHg. The abnormal movements did not recur, and the repeated blood culture was negative. A cerebrospinal fluid bacterial culture as well as a viral PCR were negative. The brain ultrasound and magnetic resonance imaging were normal. He received antibiotics for 14 days. Levetiracetam was discontinued 1 week before discharge at the post-menstrual age of 36 weeks. At the last post-discharge follow-up at 19 months of age, he had no history of recurrence of abnormal movements. He showed appropriate development for his corrected age.

## CASE 4

The fourth case is an extreme preterm male neonate born at 24 weeks gestational age in 2022, with a birth weight of 800 g. The parents were non-consanguineous and the mother was G6P3L3A2, with a history of gestational diabetes mellitus on diet. He was born via a breech vaginal delivery. His Apgar scores were 8 and 9 at 1 and 5 minutes, respectively. He was electively intubated after birth and received 2 doses of endotracheal surfactant therapy. He was extubated on day 12 of life and given non-invasive positive-pressure ventilation. However, he experienced an extubation failure within 24 hours and was reintubated on day 13 of life. On day 16 of life, he was given a dose of IV midazolam (0.1 mg/kg) for sedation, since he was agitated on the ventilator despite being on a good dose of morphine infusion. The dose was given through a peripherally inserted central catheter (PICC) line as a slow bolus, followed by a flush of 2 mL of normal saline over 2 minutes. His vital signs were stable (heart rate = 145 beats/minute, respiratory rate = 45 breaths/minute, oxygen saturation = 99%, MBP = 33 mmHg). Within seconds of the midazolam dose, he developed seizure-like clonic abnormal movements involving all 4 limbs, associated with bradycardia. He received a loading dose of phenobarbitone (20 mg/kg). The vital signs after the abnormal movement were stable (heart rate = 135 beats/minute, respiratory rate = 35 breaths/minute, oxygen saturation = 98%, MBP = 35 mmHg). Subsequently, he had no further abnormal movements. The septic work-up revealed a positive tracheal aspirate culture for *Acinetobacter baumannii*, which was treated with appropriate antibiotics. His EEG and brain ultrasound were normal and there was no seizure recurrence. He received a midazolam infusion of 10 μg/kg/hour for 72 hours without any complications. He was discharged home on nasogastric feeding due to oro-motor weakness and oral feeding difficulties.

## CASE 5

The fifth case is an extreme preterm male born at 25 weeks in 2022, twin II, conceived via *in vitro* -fertilisation, born by an emergency caesarean section to a 53-year-old mother. His Apgar scores were 8 and 9, at 1 and 5 minutes, respectively, and his birth weight was 790 g. He was intubated at birth and received a total of 2 doses of endotracheal surfactant therapy. On day 10 of life, he was desaturating and fighting against the high-frequency oscillatory mechanical ventilation despite being on a good dose of morphine infusion. A dose of IV midazolam (0.1 mg/kg) was given for sedation through a PICC line, as a slow bolus, followed by a slow flush of 2 mL of normal saline. Within a few seconds of administration of midazolam, he developed clonic seizure-like movements. His vital signs before the abnormal movement were stable (heart rate = 125 beats/minute, respiratory rate = 35 breaths/minute, oxygen saturation = 98%, MBP = 38 mmHg). He was loaded with phenobarbitone (20 mg/kg) and the seizure-like movements resolved within 2 minutes of the loading. His vital signs after the event were also stable (heart rate = 165 beats/minute, respiratory rate = 53 breaths/minute, oxygen saturation = 92%, MBP = 38 mmHg). There was no seizure recurrence and he was discharged home at the post-conceptional age of 36 weeks.

Parental consent was obtained for all neonates for publication purposes.

## Discussion

Midazolam is a commonly used sedative in NICUs.^1^^,^^7^ In addition to its sedative-hypnotic actions, midazolam also is used to treat refractory neonatal seizures and less frequently for anaesthesia.^3^ Respiratory depression and hypotension are serious well-known adverse reactions to midazolam. Paradoxical reactions to midazolam (e.g., hyperexcitability, agitation and seizures) also have been described.^5^^,^^6^

In this article, we present 5 neonates who developed seizure-like movements within seconds to minutes after the IV bolus administration of midazolam. It is worth mentioning that the cases occurred over a 4-year-period (2019–2022). No other causes of seizures could be identified by appropriate investigations performed in the 5 patients. The authors believe that the seizure-like events were induced by IV midazolam bolus administration due to the temporal association between the commencement of the IV midazolam bolus and the onset of seizure-like movements in all cases. Furthermore, no other aetiologies could be identified in any of the 5 patients. The seizure-like movements responded to anti-seizure medications, a finding that was previously described.^5^ In addition, there were no further events documented on long-term follow-up in any of the 5 patients. Moreover, the Uppsala Monitoring Centre causality assessment was checked for every patient and showed ‘probable’ for all patients except patient 3, who had a certain causality term. When the Naranjo Probability Scale was used, 4 patients were probable on the scale except for patient 3, who had definitive scoring.^7^

The distinction between seizure and seizure-like activity is challenging since both may manifest as motor activity that involves the upper and lower extremities. A seizure is a temporary disturbance in brain activity that is usually caused by increased electrical activity due to an imbalance between excitatory and inhibitory inputs. On the other hand, seizure-like activity is abnormal motor or behavioural activities that resembles a seizure. In addition, these seizure-like activities are not caused by the same abnormal brain electrical activity.^8^ A normal EEG doesn’t exclude seizure especially if the EEG was not done during the abnormal movements. Even though some of the current patients did receive anti-seizure medications to help abort the events, it is still difficult to ascertain with certainty whether the events were actual seizures or seizure-like activities.

Also, midazolam-induced seizure-like movements were described in term and preterm neonates. For example, Montenegro *et al*. reported 4 preterm neonates (34, 30, 27 and 26 weeks gestation) who developed similar clonic seizure-like movements after the administration of IV midazolam bolus for sedation.^9^ The authors ruled out possible aetiologies that could be associated with neonatal seizure, including hypoglycaemia, hypocalcaemia, infection, polycythaemia, CNS malformations and haemorrhagic or ischaemic lesions.^9^ In addition, Ozcan *et al*. described a similar adverse reaction in a preterm neonate.^10^ More recently, Gupta *et al*., described 2 preterm infants (34 and 33 weeks gestation) who developed myoclonic seizure-like movements following IV midazolam administration.^11^ In both studies, appropriate investigations were done to rule out any possible seizure aetiologies.

Moreover, this adverse reaction does not only affect preterm neonates as Zaw *et al*. reported on 3 term neonates who developed myoclonic-like abnormal movements after receiving IV midazolam. Of interest, one of them was treated with flumazenil.^12^

An explanation of why an anti-seizure medication may induce seizures has not yet been delineated. However, the rapid administration of midazolam could be the cause of the occurrence of seizure-like movements, since this adverse effect only occurred after IV bolus injection and not during continuous infusion. Van den Anker and Sauer proposed that since midazolam decreases arterial blood pressure and heart rate in preterm infants, a reduced cerebral blood flow may be the underlying pathogenesis.^6^ However, this hypothesis is a speculation and a possible direct impact on the CNS cannot be excluded. Another explanation proposed by Ishizaki *et al*. is that the abnormal movements induced by the midazolam have no epileptic origin, but rather related to a brainstem release phenomenon induced by midazolam.^13^ Since it is difficult to determine why an anti-seizure medication may induce seizures, more basic medical science research is needed to establish the cause of such a phenomenon.

Neonatal formularies recommend that midazolam boluses be administered slowly. The recommended duration of administration varies from 2–3 to 10 minutes.^14^–^17^ In our NICU, IV midazolam is administered after dilution with normal saline to 1 mg:1 mL. The dose is administered by the bedside nurse, supposedly by slow bolus as per the formulary recommendations. However, no specific duration was specified. Thus, individual variation probably existed with respect to the rate of administration. The exact duration of the administration of the dose in each of the cases could not be determined since it was not routinely documented in the electronic records. However, after observing these cases and reviewing the literature, the authors emphasised the practice of injecting IV midazolam bolus slowly over 2–3 minutes duration, followed by a slow flush of 2 mL of normal saline over 2 minutes. Since then, the authors have not observed any similar cases. Therefore, we believe that the current 5 patients received a rapid administration of the IV midazolam bolus, which played an important role in the occurrence of this rare adverse event.

## Conclusion

Although uncommon, seizure-like movements can be a side effect of midazolam IV injection. Although its underlying pathogenesis is not well determined, it may be related to the rapid administration of injection midazolam. IV midazolam is recommended to be administered with caution by slow IV injection over 2–3 minutes followed by a slow flush of normal saline, and rapid injection must be avoided. Caution should also be exercised in preterm infants, especially the extreme preterm. Neonatologists and NICU nurses should be aware of this rare adverse event in order to prevent it.
